# Neurotensin-induced miR-133α expression regulates neurotensin receptor 1 recycling through its downstream target aftiphilin

**DOI:** 10.1038/srep22195

**Published:** 2016-02-23

**Authors:** Ivy Ka Man Law, Dane Jensen, Nigel W. Bunnett, Charalabos Pothoulakis

**Affiliations:** 1 Inflammatory Bowel Disease Center, Division of Digestive Diseases, David Geffen School of Medicine, University of California at Los Angeles, California, USA; 2 Monash Institute of Pharmaceutical Sciences, ARC Centre of Excellence in Convergent Bio-Nano Science and Technology Parkville, Monash University, Australia; 3 Department of Anesthesia and Peri-operative Medicine, Monash University, Australia; 4 Department of Pharmacology and Therapeutics, University of Melbourne, Australia

## Abstract

Neurotensin (NT) triggers signaling in human colonic epithelial cells by activating the G protein-coupled receptor, the neurotensin receptor 1 (NTR1). Activated NTR1 traffics from the plasma membrane to early endosomes, and then recycles. Although sustained NT/NTR1 signaling requires efficient NTR1 recycling, little is known about the regulation of NTR1 recycling. We recently showed that NT/NTR1 signaling increases expression of miR-133α. Herein, we studied the mechanism of NT-regulated miR-133α expression and examined the role of miR-133α in intracellular NTR1 trafficking in human NCM460 colonocytes. We found that NT-induced miR-133α upregulation involves the negative transcription regulator, zinc finger E-box binding homeobox 1. Silencing of miR-133α or overexpression of aftiphilin (AFTPH), a binding target of miR-133α, attenuated NTR1 trafficking to plasma membrane in human colonocytes, without affecting NTR1 internalization. We localized AFTPH to early endosomes and the trans-Golgi network (TGN) in unstimulated human colonic epithelial cells. AFTPH overexpression reduced NTR1 localization in early endosomes and increased expression of proteins related to endosomes and the TGN trafficking pathway. AFTPH overexpression and de-acidification of intracellular vesicles increased NTR1 expression. Our results suggest a novel mechanism of GPCR trafficking in human colonic epithelial cells by which a microRNA, miR-133α regulates NTR1 trafficking through its downstream target AFTPH.

Neurotensin receptor 1 (NTR1) is a high affinity G protein-coupled receptor (GPCR) for neurotensin (NT)^1^, a 13-amino acid neuropeptide expressed in the central nervous system^2^, and the intestine, including ileum^3^ and colon^4^. NTR1 is present in colonic epithelial cells^4^,^5^,^6^,^7^ and NT/NTR1 coupling in human colonic epithelial cells activates Akt^8^,^9^, MAPK^7^,^10^,^11^,^12^ and NF-κB^7^,^9^,^10^,^13^ pathways. In human colonic epithelial cells the majority of NTR1 is internalized to Rab5a^+^ early endosomes upon NT exposure and transported back to the plasma membrane to achieve cell re-sensitization^14^. Moreover, we have shown that inhibition of NTR1 recycling from Rab5a^+^ early endosomes attenuates NT-induced proinflammatory responses in human colonic epithelial cells^14^, suggesting that NTR1 recycling and re-sensitization are required for sustained NT/NTR1 signaling activation *in vitro*. In different cells types, however, NTR1 is transported to lysosomes for degradation^15^,^16^,^17^. Therefore, the mechanisms of NTR1 re-sensitization are not well-studied.

MicroRNAs (MiRs) are small, single-stranded RNA molecules which promote translational repression or deadenylation and mRNA degradation^18^. Recently, we have shown that upon NT exposure, miR-133α and other miRs are upregulated in human colonic epithelial NCM460 cells overexpressing NTR1 (NCM460-NTR1)^8^. Reducing miR-133α levels in NCM460-NTR1 cells attenuated NT-induced MAPK and NF-κB activation and cytokine production^19^. MiR-133α has been shown to regulate ERK, PI3K/Akt and p53 signaling pathways^20^,^21^,^22^,^23^,^24^ by directly targeting transcripts related to these pathways. Interestingly, gene silencing of aftiphilin, a novel target of miR-133α associated with intracellular trafficking^25^,^26^ and secretion^27^, also promotes proinflammatory responses in human colonic epithelial cells^19^. Taken together, we hypothesize that miR-133α mediates NTR1-associated signaling pathways by modulating intracellular NTR1 trafficking. Here we present evidence that the miR-133α/AFTPH axis controls intracellular NTR1 trafficking in human colonic epithelial cells and identified zinc finger E-box binding homeobox 1 (ZEB1) as a negative transcriptional regulator of NT-induced miR-133α expression. Our results also show that the miR-133α/AFTPH axis modulates the expression of proteins along the endosome and trans-Golgi network (TGN) transport pathways, representing a likely mechanism for regulating trafficking of NTR1 in human colonic epithelial cells.

## Results

### ZEB1 is a negative transcriptional regulator of miR-133α

**Figure 1:**
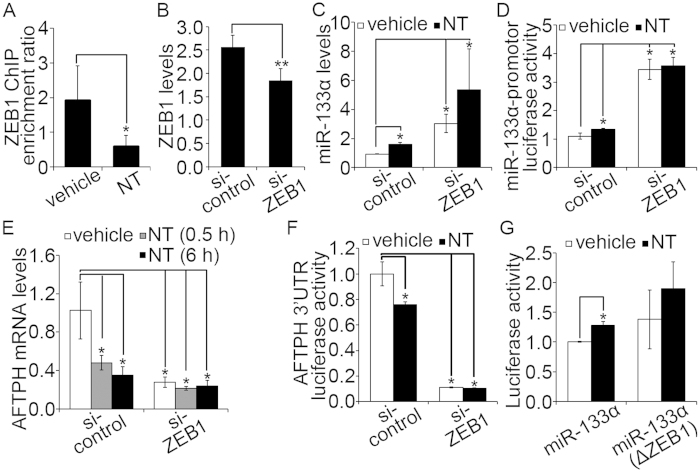
ZEB1 was a negative transcription regulator of NT-induced miR-133α expression in human colonic NCM460-NTR1 cells. (**A**) NT exposure (100 nM, 1 hr) reduced ZEB1 nuclear binding as evaluated in ChIP assay with nuclear extracts from NCM460-NTR1 cells incubated with NT and its vehicle control. (**B**) Expression of ZEB1 was reduced in NCM460-NTR1 cells transfected with si-ZEB1 as examined by RT-PCR when compared with cells transfected with scramble controls. (**C**) ZEB1 gene silencing in NCM460-NTR1 cells increased miR-133α levels at unstimulated condition as examined by RT-PCR when compared with cells transfected with scramble controls. (**D**) Increased miR-133α promoter-driven luciferase activities were detected from NCM460-NTR1 cells transiently transfected with si-ZEB1 when compared with cells transfected with scramble controls at unstimulated condition. (**E**) ZEB1 gene silencing reduced AFTPH levels were detected by RT-PCR in NCM460-NTR1 cells when compared with cells transfected to scramble controls. (**F**) Reduced AFTPH 3′UTR-driven luciferase activities were detected in ZEB1 gene-silenced NCM460-NTR1 cells when compared to cells transfected with scramble controls, as measured in luciferease assay. (**G**) No significant increase in luciferase activities was observed in cells transfected with plasmid expressing miR-133α promoter without ZEB1 binding site in the presence NT exposure (100 nM, 1 hr), when compared to those transfected with plasmid with ZEB1 binding site. *p < 0.05, **p < 0.01 when compared with control.

We have recently reported that incubation of NCM460-NTR1 cells with NT in human colonocytes increases expression of miR-133α^8^,^19^. To examine the molecular mechanism by which NT induces miR-133α upregulation, the genomic sequence of 2000 bp upstream to the transcription start site (TSS) of miR-133α was analyzed by the online transcription binding site prediction software, Transcription Element Search System (TESS) (http://www.cbil.upenn.edu/cgi-bin/tess/tess). A transcription binding site (CTGTTTCAC) for ZEB1 was found upstream to miR-133α at position −1150 bp to −1142 bp. We first validated binding of ZEB1 in NT-stimulated human colonic epithelial NCM460-NTR1 cells by immunoprecipitating nuclear extracts from control and NT-exposed cells with a ZEB1antibody and performing chromatin immunoprecipitation (ChIP). Our results showed ZEB1 binding was reduced upon NT stimulation (Fig. 1A). We next knocked down ZEB1 expression by siRNA in NCM460-NTR1 cells and exposed them to 100 nM NT (1 hr). RT-PCR analysis suggested that expression of ZEB1 in cells transfected with si-ZEB1 was significantly reduced (Fig. 1B, *p* < 0.01). Upon ZEB1 knock-down, NT did not significantly increase miR-133α levels (Fig. 1C) or miR-133α promoter activity (Fig. 1D), although in cells transfected with scrambled siRNA, both increased significantly following NT treatment (Fig. 1C,D, *p* <  0.05). In addition, basal transcription and promoter activity of miR-133α in ZEB1-silenced cells were significantly higher compared to controls (Fig. 1C,D, *p* <  0.05). In our previous study, we show that miR-133α knock-down in NCM460-NTR1 cells blocked NT-induced reduction in AFTPH mRNA expression and AFTPH 3′UTR-driven luciferase activities^19^, implicating that miR-133α regulates AFTPH expression by destabilizing AFTPH mRNA transcript. In the present study, we measured expression of AFTPH following ZEB1 gene-silencing. NT stimulation reduced AFTPH transcription levels (Fig. 1E, *p* <  0.05) and AFTPH 3′UTR-driven luciferase activity (Fig. 1F, *p* <  0.05) in controls, but not in ZEB1-silenced cells. Basal AFTPH luciferase activity and transcription levels were reduced significantly in ZEB1-silenced cells compared to cells transfected with scrambled siRNA (Fig. 1E,F, *p* < 0.05). To examine whether miR-133α promoter activity is associated with ZEB1 binding, a luciferase reporter without the ZEB1 binding sequence was constructed. As shown in Fig. 1G, increased miR-133α promoter-driven luciferase activity in response to NT was abolished in cells transfected with a miR-133α promoter with deleted ZEB1 binding site. Therefore, ZEB1 acts as a negative transcriptional regulator in NT-associated miR-133α transcription that mediates increased miR-133α expression in response to NT.

### MiR-133α is involved in intracellular trafficking of NTR1 after NT exposure

**Figure 2:**
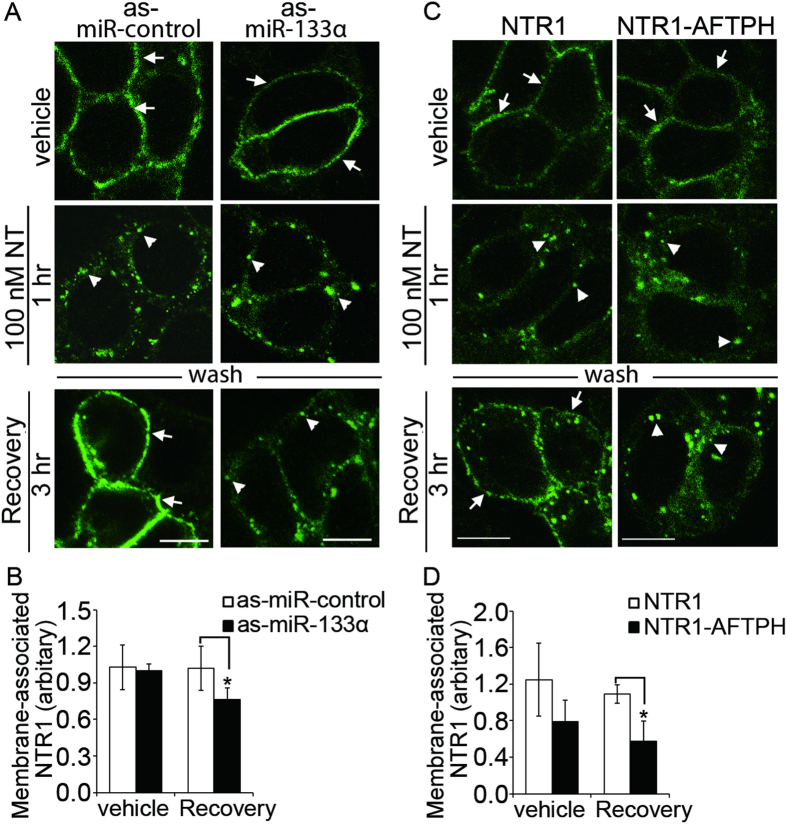
MiR-133α/AFTPH axis regulated NTR1 trafficking to plasma membrane during recovery in human colonic epithelial cells. (**A**) NCM460-NTR1 cells transfected with as-miR-133α and as-miR-control were exposed to NT (100 nM, 1 hr), washed and replenished with NT-free media (recovery). Trafficking of NTR1 to plasma membrane was attenuated in miR-133α knocked-down NCM460-NTR1 cells, as visualized using immunocytochemistry. (arrow: membrane-bound; arrowhead: vesicle-bound) Scale bars, 10 μm (**B**) Reduced membrane-bound NTR1 was present in the miR-133α-silenced cells after recovery when compared with control cells, as measured in biotinylation assay. *p < 0.05 when compared to as-miR-control-transfected cells. (**C**) NCM460-NTR1 (NTR1) cells and cells overexpressing NTR1 and AFTPH (NTR1-AFTPH) were exposed to NT, washed and allowed to recover as described in (**A**). Trafficking of NTR1 to plasma membrane was attenuated in NCM460-NTR1-AFTPH cells as visualized using immunocytochemistry when compared with NCM460-NTR1 cells. (arrow: membrane-bound; arrowhead: vesicle-bound) Scale bars, 10 μm (**D**) Reduced membrane-bound NTR1 was observed after recovery in NCM460-NTR1-AFTPH cells when compared to NCM460-NTR1 cells in biotinylation assay. *p < 0.05 when compared to NCM460-NTR1 cells.

MiR-133α has been associated with myocyte development and hypertrophy^28^,^29^,^30^, fibrosis^31^,^32^, and oncogenesis^20^,^21^,^33^,^34^,^35^,^36^,^37^. We recently showed that miR-133α gene silencing attenuates colonic inflammation during experimental colitis, possibly by reducing NT-mediated proinflammatory response in colonic epithelial cells^19^. NT, through NTR1, activates proinflammatory signaling pathways in colonic epithelial cells^7^,^9^,^10^,^13^ and this signaling activation is, at least, partially regulated by efficient recycling of NTR1 to the plasma membrane^14^. Therefore, we hypothesized that NT-upregulated miR-133α might also contribute to intracellular trafficking of NTR1. To localize NTR1 during receptor trafficking in the presence or absence of miR-133α, human colonic epithelial NCM460-NTR1 cells were transfected with antisense (as)-miR-133α or its control and exposed to 100 nM NT (1 hr) in serum-free media to allow NTR1 internalization. The cells were then washed and replenished with NT-free media for 3 hr to allow NTR1 trafficking to the cell surface (recovery). NTR1 cells treated with vehicle control remained on the plasma membrane, while NT (100 nM) exposure internalized NTR1 (Fig. 2A). Importantly, internalization of NTR1 was not affected by miR-133α gene-silencing. However, miR-133α downregulation attenuated trafficking of NTR1 to the plasma membrane by retaining NTR1 within intracellular vesicles, while the majority of NTR1 in cells transfected with control as-miR was localized to the plasma membrane 3 hr after removal of NT from the media (Fig. 2A). To confirm this, we performed a biotinylation assay on NCM460-NTR1 cells after recovery, followed by NTR1-specific ELISA to quantify NTR1 localized to the cell surface. As-miR-133α treatment significantly reduced the presence of NTR1 on plasma membrane after recovery (1.0 ± 0.18 vs 0.8 ± 0.10, *p* < 0.05), when compared to control as-miR-treated cells (Fig. 2B). These results suggest that knock-down of miR-133α attenuates NTR1 trafficking to plasma membrane in human colonic epithelial cells.

### MiR-133α regulates intracellular trafficking of NTR1 through its binding target, aftiphilin

We have previously identified a novel miR-133α binding target, aftiphilin (AFTPH), which is downregulated following NT exposure of human colonic epithelial NCM460-NTR1 cells^19^. The function of AFTPH in intestinal epithelial cells is not known. Previous studies suggested that AFTPH knock-down promoted the recycling of endocytosed transferrin to cell periphery in HeLa cells^26^ and dysregulated exocytosis of Weibel-Palade bodies in endothelial cells without affecting the process of maturation of the granules^27^. To examine the function of AFTPH in intracellular trafficking of NTR1, NCM460-NTR1 cells overexpressing lentivirus – transduced AFTPH (NCM460-NTR1-AFTPH) were generated and exposed to NT (100 nM) followed by recovery for 3 hr. RT-PCR analysis showed that AFTPH expression in NCM460-NTR1-AFTPH cells was significantly increased (Suppl. Fig. 1, *p* < 0.05). Furthermore, since exogenous AFTPH lacks the original 3′UTR, its expression level should not be reduced upon NT stimulation. We found that NTR1 was localized on the plasma membrane of NCM460-NTR1-AFTPH cells treated with vehicle control (Fig. 2C). Thus, similar to miR-133α silencing, overexpression of AFTPH did not affect NTR1 internalization or expression of NTR1 on the plasma membrane. However, without AFTPH downregulation during NT exposure, trafficking of NTR1 to the plasma membrane was attenuated when compared to NCM460-NTR1 cells (Fig. 2C). Results from biotinylation assays also confirmed that reduced presence of NTR1 on the plasma membrane after recovery (1.1 ± 0.10 vs 0.6 ± 0.22, *p* < 0.05), when compared to NCM460-NTR1 cells (Fig. 2D). In addition, NTR1 internalization and recycling were also examined in NCM460–NTR1 cells transfected with si-AFTPH and its scrambled si-control. As expected, NTR1 (localized in intracellular vesicles) was further reduced 3 hr after recovery compared to si-control transfected cells (Suppl. Fig. 2A). Our biotinylation assay also showed that membrane-bound NTR1 was increased in si-AFTPH-transfected cells after recovery (1.3 ± 0.61 vs 2.8 ± 0.58, *p* < 0.05) when compared to si-control-transfected cells (Suppl. Fig. 2B). Taken together, absence of AFTPH downregulation during NT exposure attenuates NTR1 trafficking to the plasma membrane in human colonic epithelial cells.

### AFTPH overexpression modulates intracellular NTR1 trafficking upon NT exposure

**Figure 3:**
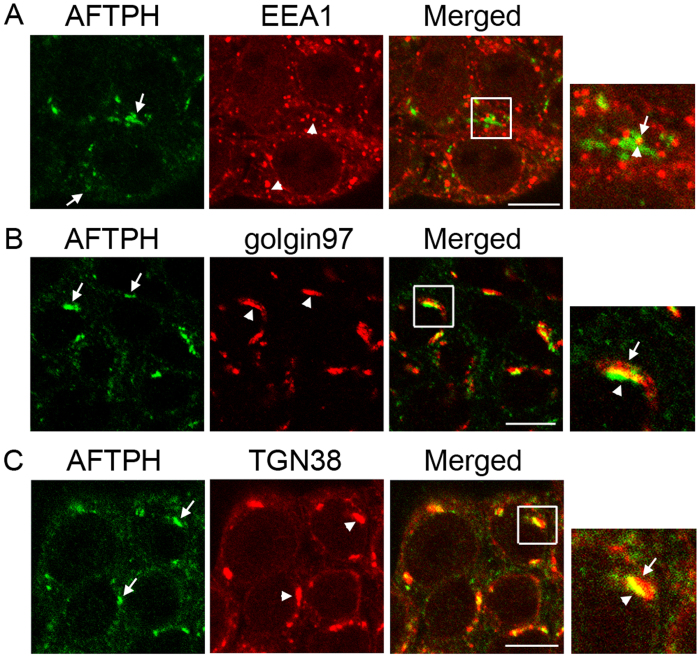
AFTPH was localized in both early endosomes and *trans*-Golgi network in human colonic epithelial cells. (**A**) Endogenous AFTPH (arrows) was co-localized with EEA1 (arrowheads), an early endosome marker, in NCM460-NTR1 cells. Majority of the endogenous AFTPH (arrows) displayed a peri-nuclear localization and was co-localized with trans-Golgi network markers (**B**) golgin 97 (arrowheads) and (**C**) TGN38 (arrowheads) in NCM460-NTR1 cells, as visualized in immunocytochemistry. Scale bars, 10 μm.

Since as discussed above AFTPH is a novel miR-133α downstream target in human colonic epithelial cells exposed to NT^19^, we examined the role of AFTPH in NTR1 trafficking in NCM460-NTR1 cells. AFTPH has been localized in TGN^25^ and early endosomes^26^ in different cell types. In the present study, without NT stimulation, immunocytochemical analysis showed that a small number of AFTPH exhibited cytosolic localization and co-localized with EEA1, a marker for early endosomes (Fig. 3A). However, the majority of AFTPH displayed a peri-nuclear localization and was co-localized with the TGN markers golgin 97 and TGN38, (Fig. 3B,C). These results suggest that AFTPH is localized in both early endosomes and TGN in non-stimulated human colonic epithelial cells.

**Figure 4:**
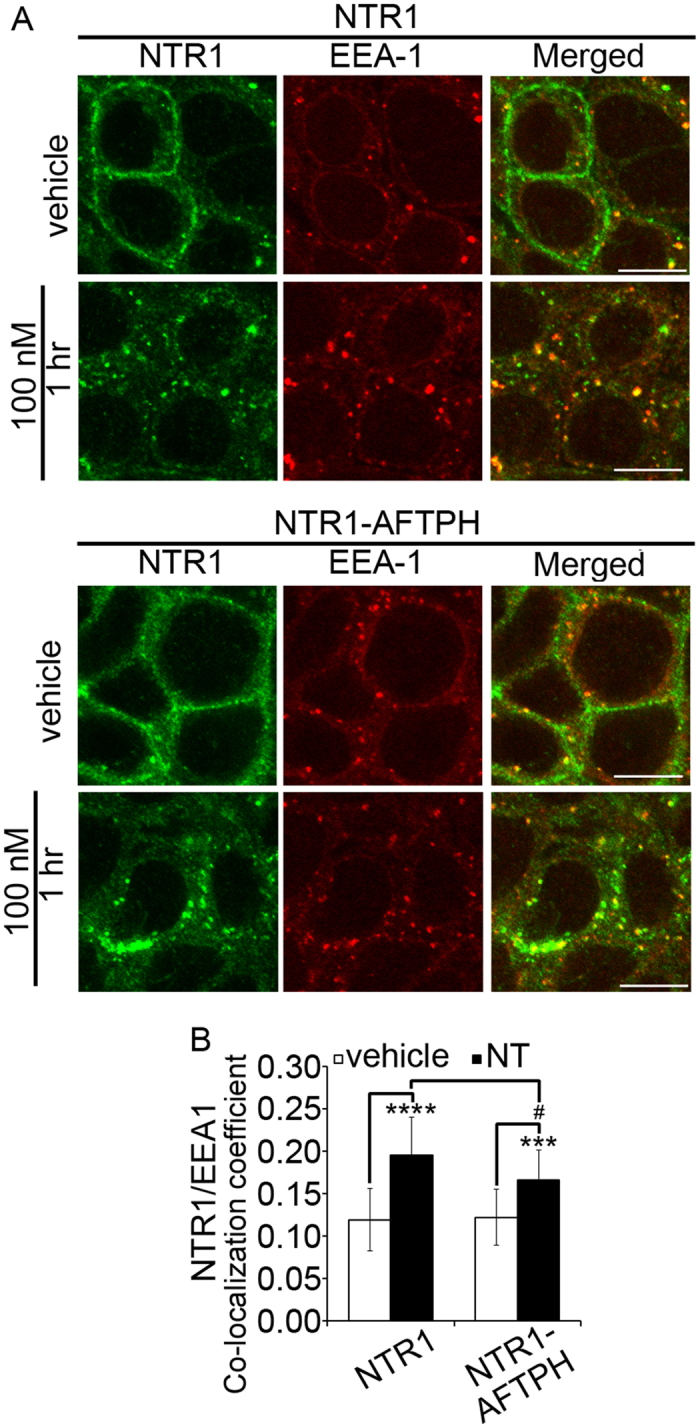
AFTPH overexpression reduced NTR1 localization in early endosomes upon NT stimulation in human colonic epithelial cells. (**A**) Cells with AFTPH overexpression (NTR1-AFTPH) showed reduced co-localization of internalized NTR1 and EEA-1, an early endosome marker, upon NT stimulation (100 nM, 1 hr), when compared with NCM460-NTR1 cells as examined in immunocytochemistry. Scale bars, 10 μm. (**B**) Co-localization of NTR1 and EEA1 was increased upon NT stimulation in both cell lines when compared with the unstimulated condition, as examined by quantitative image analysis. However, co-localization of internalized NTR1 and EEA1 was reduced in NCM460-NTR1 cells overexpressing AFTPH upon NT stimulation when compared with NCM460-NTR1 cells. ***p < 0.005, ****p < 0.0001 when compared to cells in unstimulated conditions. ^#^*p* < 0.05 when compared to NCM460-NTR1 cells without AFTPH overexpression.

We have shown that translocation of NTR1 to early endosomes is essential for internalized NTR1 to be transported back to the plasma membrane^14^. In addition, AFTPH knock-down prevents trafficking of internalized transferrin from early endosomes to recycling endosomes, and thus leads to the accumulation of transferrin in early endosomes^26^. Therefore, we next examined whether AFTPH overexpression affected NTR1 translocation to early endosomes. NCM460-NTR1 and NCM460-NTR1-AFTPH cells were exposed to NT (100 nM, 1 hr) and NTR1 and EEA1 were localized by immunocytochemistry. NTR1 was localized on the plasma membrane under unstimulated condition and internalized upon stimulation in both cell types (Fig. 4A). NTR1/EEA1 co-localization under both conditions in the two cell lines was then quantified by analyzing the images from the immunocytochemistry experiments. As expected, NT exposure increased NTR1/EEA1 co-localization in both cell lines when compared to unstimulated cells (Fig. 4B, *p* < 0.001). More importantly, AFTPH overexpression reduced co-localization of NTR1 with EEA1 (0.20 ± 0.04 vs 0.17 ± 0.04, *p* < 0.05) when compared to NCM460-NTR1 cells (Fig. 4B). Therefore, overexpression of AFTPH, a TGN-localized protein, reduces NTR1 translocation to early endosomes during NT stimulation.

### MiR-133α/AFTPH axis modulates expression of proteins in endosomes and TGN

**Figure 5:**
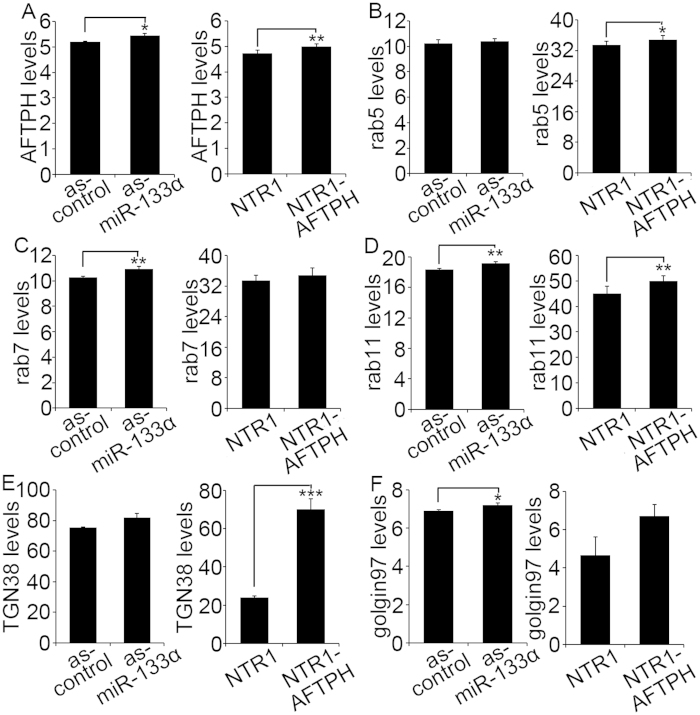
MiR-133α/AFTPH axis regulated expression of proteins related to endosome and trans-Golgi network trafficking pathways. (**A**) NCM460-NTR1 cells transfected with as-miR-133α and NCM460-NTR1-AFTPH cells showed increased AFTPH expression in In-cell ELISA assay, when compared to their respective controls. (**B**) Rab5 expression was increased in NCM460-NTR1-AFTPH cells, when compared to NCM460-NTR1 cells. (**C**) Rab7 expression was increased in miR-133α gene-silenced NCM460-NTR1 cells when compared to the controls. (**D**) Rab11 expression was increased in both NCM460 cells transfected with as-miR-133α and NCM460-NTR1-AFTPH cells when compared to their respective controls. (**E**) Increase in TGN38 expression was observed in NCM460-NTR1-AFTPH cells when compared with controls. (**F**) Golgin97 expression was increased in NCM460-NTR1 cells transfected with as-miR-133α, when compared with controls. *p < 0.05, **p < 0.01, ***p < 0.005 when compared with respective controls.

Next, we studied the effect of miR-133α and AFTPH overexpression on expression of proteins along endosome trafficking pathways. Transiently miR-133α gene- silenced NCM460-NTR1 cells and NCM460-NTR1-AFTPH cells were fixed and expression of rab5 (early endosome), rab7 (late endosome), and rab11 (recycling endosome) were determined by In-cell ELISA and compared with their respective controls. We first validated this protein quantification method by showing miR-133α gene-silencing and AFTPH overexpression increased AFTPH expression (Fig. 5A). Next, we showed that rab7 and rab5 expression were increased upon miR-133α gene-silencing and AFTPH overexpression, respectively (Fig. 5B,C). More importantly, rab11 expression was significantly increased in both NCM460-NTR1 cells transfected with as-miR-133α and NCM460-NTR1-AFTPH cells when compared to their respective controls (Fig. 5D). These data suggest that the miR-133α/AFTPH axis modulates the expression of rab11 from recycling endosomes. However, expression of other endosome markers was also increased, but the results were not statistically significant (Fig. 5B,C). The differential expression levels of rab5 and rab7 might be due to differential regulation of expression of genes targeted by miR-133α, other than AFTPH.

Since AFTPH was also localized in TGN (Fig. 3), we next studied the expression of TGN markers in human colonic epithelial cells. Results from In-cell ELISA suggested that expression of golgin97 and TGN38 was significantly increased upon miR-133α gene-silencing and AFTPH overexpression, respectively (Fig. 5E,F). Thus, miR-133α knock-down and AFTPH overexpression results in increased expression of markers of recycling endosomes and TGN.

### MiR-133α/AFTPH axis acts on a Bafilomycin A1-sensitive trafficking network

**Figure 6:**
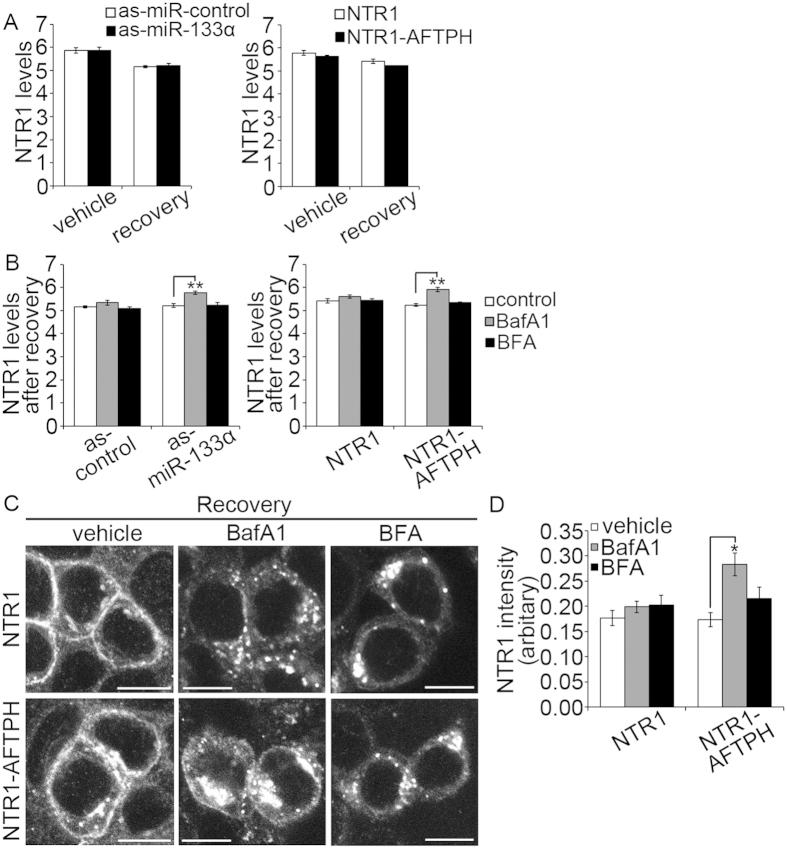
AFTPH overexpression increased NTR1 levels in human colonic epithelial cells treated by Bafilomycin A1. (**A**) MiR-133α gene silencing and AFTPH overexpression in NCM460-NTR1 cells did not affect NTR1 levels in human colonic epithelial cells when compared with their respective controls under the same treatment (unstimulated or upon 3 hr after recovery), as examined by In-cell ELISA. (**B**) Treatment with Bafilomycin A1 (BafA1, 100 nM), a specific vacuolar H^+^-ATPase inhibitor, increased NTR1 levels in as-miR-133α-transfected or AFTPH-overexpressing cells when compared to their respective controls. Similar effects were not observed in cells treated with Brefeldin A (BFA, 5 μg/mL). **p < 0.01 when compared with cells without incubating with inhibitors during recovery. (**C**) Bafilomycin A1 and Brefeldin A treatment during recovery blocked NTR1 recycling in both NCM460-NTR1 cells and their AFTPH-overexpressing counterparts when compared to their respective controls, as visualized in immunocytochemistry. Scale bars, 10 μm. (**D**) Intracellular NTR1 expression was increased in NCM460-NTR1-AFTPH cells treated with Bafilomycin A1, when compared to NCM460-NTR1 cells under the same treatment as examined in quantitative image analysis. Incubation with Brefeldin A during recovery did not generate similar effect on the two cell lines. *p < 0.05 when compared with cells treated with vehicle controls.

We have shown that miR-133α knock-down and AFTPH overexpression retained NTR1 in the cytosol (Fig. 1) during recovery and upregulated protein expression in endosomes and TGN (Fig. 5). On the other hand, studies in other cell types have suggested that NTR1 undergoes lysosome degradation^15^,^16^,^17^,^38^. Therefore, we next examined whether miR-133α or AFTPH overexpression contributed to NTR1 degradation by examining NTR1 levels in NCM460-NTR1 cells with either silenced miR-133α or overexpressing AFTPH. Cells were exposed to NT (100 nM, 1 hr), allowed to recover in NT-free media for 3 hr, and then fixed for NTR1 levels quantification by In-cell ELISA. Our results suggested that miR-133α and AFTPH levels did not affect NTR1 levels in either unstimulated cells or in cells after recovery from NT exposure (Fig. 6A). Our findings suggest that AFTPH overexpression during recovery promotes NTR1 retention in the cytosol, without affecting NTR1 degradation in human colonic epithelial cells.

Next, we examined whether attenuation of NTR1 trafficking to cell membrane was related to de-acidification of intracellular vesicles or impaired TGN functions. Bafilomycin A1 (BafA1) is a specific vacuolar H^+^-ATPase inhibitor^39^, which can alter the acidic conditions^40^,^41^,^42^,^43^ in recycling endosomes and TGN^44^ and inhibit clathrin-coated vesicle (CCV) formation and clathrin-mediated internalization^45^. Of note, in our previous study, NCM460-NTR1 cells treated with BafA1 (100 nM) during recovery show retention in NTR1 in the cytosol^14^. On the other hand, although NT has been localized in TGN^16^, the role of TGN in NTR1 trafficking is largely unknown. Here, we have treated as-miR-133α-transfected NCM460-NTR1 cells and NCM460-NTR1-AFTPH cells and their respective controls with BafA1 (100 nM) and Brefeldin A (BFA, 5 μg/mL) during recovery. BFA is a fungal metabolite redistributing TGN proteins to endoplasmic reticulum and early endosomes depending on the cell type^46^,^47^. DMSO alone, the solvent for BafA1 and BFA, was used as a control treatment. Expression levels and intracellular distribution of NTR1 in NCM460-NTR1 cells with or without AFTPH overexpression were measured and visualized by In-cell ELISA and immunocytochemistry, respectively. As shown in Fig. 6B, BafA1 and BFA treatment did not alter NTR1 levels in NCM460-NTR1 cells transfected with scrambled control or in non-transfected NCM460-NTR1 cells after recovery. However, BafA1 treatment during recovery increased NTR1 levels in miR-133α gene-silenced NCM460-NTR1 cells and NCM460-NTR1-AFTPH cells (Fig. 6B). BFA treatment did not alter NTR1 levels in any experimental group. We further verified this observation with immunocytochemistry in NCM460-NTR1 cells and NCM460-NTR1-AFTPH cells using NTR1-specific antibodies. Our results showed that the majority of NTR1 in NCM460-NTR1 cells were present at the plasma membrane, while those in AFTPH-overexpressing cells were partially retained in the cytosol (Fig. 6C). On the other hand, NTR1 recycling was largely inhibited by BFA treatment in both cell lines (Fig. 6C). BafA1 treatment also inhibited NTR1 recycling in both cell lines, similar to our previous observation^14^ (Fig. 6C). In addition, our results suggested that NTR1 aggregated in the cytosol under BafA1 treatment during recovery in NCM460-NTR1-AFTPH cells, but not in NCM460-NTR1 cells (Fig. 6C), while, NTR1 distribution was similar in both cell lines under BFA treatment. Similar to the data from In-cell ELISA, quantitative image analysis on signals from NTR1 suggested that NTR1 expression was increased in NCM460-NTR1-AFTPH cells under BafA1 treatment only (Fig. 6D, *p* < 0.05), but not in NCM460-NTR1 cells. Thus, although BafA1 treatment during recovery did not change NTR1 levels in control cells, BafA1 treatment, but not BFA, increased NTR1 levels in both as-miR-133α-transfected NCM460-NTR1 cells and NCM460-NTR1-AFTPH cells. Taken together, our results indicate that AFTPH is involved in BafA1-sensitive intracellular trafficking pathway during NTR1 translocation.

## Discussion

**Figure 7:**
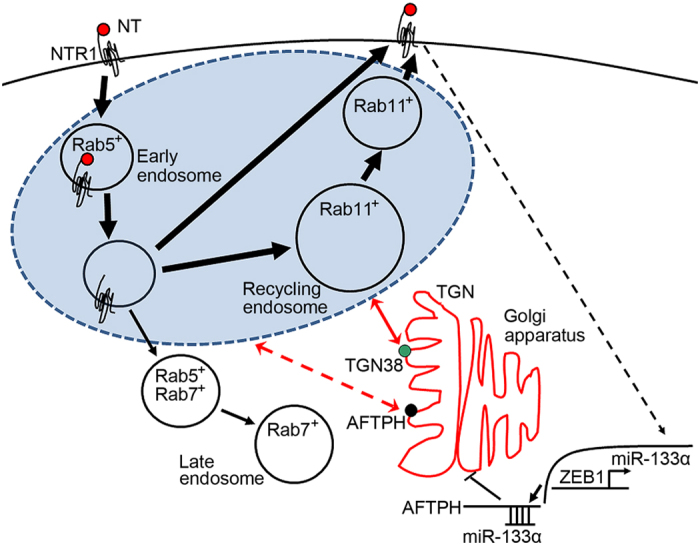
A schematic diagram showing the proposed mechanism for intracellular NTR1 trafficking regulated by NT-mediated miR-133α/AFTPH axis in human colonic epithelial cells. NT/NTR1 coupling induced detachment of negative transcription regulator ZEB1 from the promoter region of miR-133α in nucleus in human colonic epithelial cells. Increased miR-133α transcription reduced endogenous AFTPH levels in TGN *in vitro*, and promoted intracellular NTR1 trafficking to cell membrane. Our results suggested failure to reduce endogenous AFTPH levels after NT stimulation caused NTR1 retention in cytoplasm, which coincided with increased Rab11 expression in endosome trafficking pathway.

NT activates proliferative^8^,^9^,^11^,^12^ and pro-inflammatory^7^,^9^,^10^,^13^ signaling pathways in human colonic epithelial cells through its high affinity GPCR, NTR1^1^. Recently, we have shown that NT/NTR1 coupling increases miR-133α levels in human colonic epithelial cells and knock-down of miR-133α downregulates NT-induced proinflammatory signaling *in vitro*^8^ through its downstream target AFTPH^19^. On the other hand, we have also shown that sustained NTR1 signaling is associated with efficient trafficking of NTR1 to the cell surface after NT stimulation in human colonic epithelial cells^14^. Taken together, these results suggested that NT-induced miR-133α expression may be involved in NTR1 trafficking *in vitro*. In this current study, we show that miR-133α expression is negatively regulated by the transcription factor ZEB1 via a ZEB1 binding site on its promoter upon NT stimulation in human colonic epithelial cells. We further showed that the NT-driven miR-133α/AFTPH axis regulates NTR1 trafficking and expression of proteins involved in endosomal trafficking and NTR1 intracellular destination following ligand exposure (Fig. 7).

Using a combination of bioinformatic analysis, RT-PCR, ChiP and luciferase assays, we identified ZEB1 as a NT-driven negative transcriptional regulator of miR-133α in human colonic epithelial cells (Fig. 1). Our results reveal that ZEB1 directly binds to the miR-133α promoter region and suppresses miR-133α expression acting as a negative transcription regulator (Fig. 1). Studies in skeletal muscle differentiation suggest that other transcription factors, such as MyoD and myogenin bind to the miR-133α promoter and increase miR-133α expression^28^,^29^, while the ecotropic virus integration site 1 (EVI1) directly binds to the miR-133α promoter in human erythroblast HEL cells^48^. The role of ZEB1 as transcription repressor in the present study supports its putative role as a transcriptional repressor during epithelial-mesenchymal transition (EMT)^49^,^50^ and dedifferentiation^51^. Of interest, colonic epithelial miR-133α expression is associated with colon cancer development^20^,^21^,^24^,^33^,^34^,^35^. Since high NTR1 expression was observed in both inflamed colonic tissues^4^,^5^,^6^ and in colonic cancer cells^8^, suppression of miR-133α expression by ZEB1/miR-133α binding in human colonic epithelial cells may play a role in the NTR1/miR-133α^19^ interactions during colonic inflammation and early cancer development through NTR1 re-sensitization *in vitro* and *in vivo*.

Although intracellular NTR1 trafficking has been studied extensively^14^,^15^,^16^,^52^, the role of miRs in trafficking of NTR1 or other GPCRs has not been studied. We show that blocking NT-induced miR-133α upregulation in human colonic epithelial cells increases NTR1 retention in the cytoplasm as observed by confocal microscopy and biotinylation assays (Fig. 2A,B). Moreover, we have also show that overexpression of AFTPH, a miR-133α target^19^, also attenuates NTR1 trafficking back to plasma membrane after NT exposure (Fig. 2C,D). Our data suggested that since miRs regulate mRNA stability^53^ and protein translation^54^,^55^, miR expression induced by GPCR activation may present a novel regulatory pathway for GPCR recycling on an epigenetic level.

Previous studies demonstrate that AFTPH is localized in clathrin-coated vesicles (CCV)^25^,^26^, TGN^25^ and early endosomes^26^ in neurons and HeLa cells. Our present study extended these observations by showing that the majority of AFTPH are localized in the TGN in human colonic epithelial cells (Fig. 3B,C). Furthermore, AFTPH is also localized in the early endosomes (Fig. 3A), which may be explained by the interaction of AFTPH with the AP-1 complex, which is localized in endosomes^25^,^26^. Functionally, AFTPH is associated with exocytosis in endothelial cells^27^. During transferrin recycling, knocking down AFTPH promotes the transport of internalized transferrin to early endosomes^26^. In our study, the localization of NTR1 remains cytosolic after recovery in human colonic epithelial cells overexpressing AFTPH (Fig. 2C,D) while AFTPH knocking down promotes trafficking of NTR1 to the plasma membrane (Suppl. Fig. 2). More importantly, AFTPH overexpression reduced the co-localization of internalized NTR1 and EEA1, an early endosome marker (Fig. 4), in human colonic epithelial cells. The above observations and those in transferrin recycling^26^ suggest that AFTPH knock-down induced by NTR1/miR-133α regulation^19^ suppresses the trafficking of NTR1 from early endosomes to recycling endosomes, thus promoting the rapid recycling by NTR1 from early endosomes back to the plasma membrane^26^. Cell desensitization to a ligand-stimulant depends on the efficiency of receptor internalization, while cell re-sensitization is partially achieved by transporting unbound receptors back to plasma membrane. In human colonic epithelial cells, NT-induced proinflammatory responses are attenuated by downregulation of miR-133α, but promoted by AFTPH gene silencing^19^. Therefore, part of NT/NTR1 signaling might promote trafficking of internalized NTR1 to the plasma membrane through a miR-133α/AFTPH axis, leading to NTR1 re-sensitization.

In addition, AFTPH overexpression by both miR-133α knock-down and stable AFTPH overexpression increased expression of rab11 (recycling endosome marker), and TGN38 and golgin97 (TGN markers) in human colonocytes (Fig. 5) without affecting NTR1 expression levels during recovery (Fig. 6A). TGN38 and golgin97 translocate between endosomes and TGN^56^,^57^,^58^,^59^, therefore, our results suggests AFTPH overexpression modulates NTR1 trafficking through upregulating expression of proteins involved in trafficking between endosomes and TGN during recovery. We also demonstrated the importance of TGN to NTR1 trafficking to the plasma membrane during recovery by showing NTR1 retention in cytoplasm in human colonic epithelial cells with TGN structure and function disrupted by BFA treatment^47^,^60^ (Fig. 6C). Interestingly, BFA-induced inhibition of NTR1 trafficking to the plasma membrane during recovery did not affect NTR1 expression in human colonic epithelial cells regardless of AFTPH expression levels. However, de-acidification of endosomes and TGN by BafA1, a specific vacuolar H^+^-ATPase inhibitor^39^, increased NTR1 levels in cells overexpressing AFTPH (Fig. 6B,C). Since the low pH environment of TGN and recycling endosomes is maintained by functional H^+^-ATPase^44^, the accumulation of NTR1 in AFTPH-overexpressing cells suggests that AFTPH may be associated with NTR1 degradation when endosome and TGN transport are impeded. Of note, loss of endosomal acidification may also prevent NTR1 recycling by attenuating the dissociation of NT/NTR1 complex in early endosomes^14^, which, in turn, leads to dysregulation in NTR1 recycling. In contrast, since BFA redistributes TGN vesicles to endoplasmic reticulum and early endosomes^46^,^47^, our results imply that AFTPH may not be involved in structural maintenance of TGN in human colonic epithelial cells, as observed in neurons^25^.

In summary, NT/NTR1 coupling induces the dissociation of ZEB1, a negative transcription regulator, to miR-133α promoter, leading to upregulation of miR-133α. MiR-133α and its downstream target AFTPH, in turn, regulate the intracellular trafficking of the internalized NTR1 and thereby regulating the NTR1 recycling efficiency and re-sensitization in human colonic epithelial cells. AFTPH overexpression increases expression of proteins participating in endosome and TGN transport (Fig. 7). Our results suggest that NT/ZEB1/miR-133α/AFTPH signaling may represent a novel epigenetic regulatory network involved in NTR1 trafficking.

## Methods

### Materials

NT was from Bachem Americas, Inc (Torrance, CA). Bafilomycin A1 and Brefeldin A (both dissolved in DMSO) were from Santa Cruz Biotechnology. Cell culture medium M3:D was from INCEL Corp. (San Antonio, TX). Antisense-miR-133α (as-miR-133α) and its negative control (as-miR-control), TaqMan^®^ probe against AFTPH and ZEB1, TaqMan^®^ Universal PCR Master Mix (2×), Lipofectamine 2000, Lipofectamine™ RNAiMAX, TRIzoL, Pierce™ Blocking Buffer were from Life Technologies (Carlsbad, CA). si-RNA against ZEB1 (si-ZEB1), and their negative control (si-Control) were from Santa Cruz Biotechnology. EZ-link^®^ Sulfo-NHS-LC Biotin and Pierce Agarose ChIP kit were from Thermo Scientific (Rockford, IL). Glutathione was from Fisher Scientific (Rockford, IL). QuikChange II XL site-directed mutagenesis Kit was from Agilent Technologies (Santa Clara, CA). Dual-luciferase reporter assay system, pGL3-Basic and pRL-TK were from Promega BioSciences (St. Luis, CA). AFTPH 3′ UTR luciferase reporter plasmid was from Switchgear Genomics (Carlsbad, CA). hsa-miR-133a-5p LNA™ PCR primer set, Universal cDNA Synthesis Kit II and ExiLENT SYBR® Green master mix were from Exiqon (Vedbaek, Denmark). IRDye® 800 CW Donkey anti-Mouse IgG, IRDye® 680RD Donkey anti-Goat IgG, CellTag™ 700 Stain are from LI-COR Biosciences (Lincoln, NE). Antibodies used in this study include: Goat polyclonal antibodies against AFTPH (sc-167055) and NTR1 (sc-7596); rabbit polyclonal antibodies against early endosomal antigen 1 (EEA1, sc-33585), trans-Golgi network protein 2 (TGN38, sc-33783), Rab5a (sc-309), Rab7 (sc-10767), Rab11 (sc-9020); and mouse monoclonal antibody against Golgi-associated protein golgin A1 (golgin 97, sc-59820) were purchased from Santa Cruz Biotechnology (Santa Cruz, CA). Bovine anti-goat IgG-FITC (sc-2348), bovine anti-rabbit IgG-R (sc-2367) and bovine anti-mouse IgG-R (sc-2368) are from Santa Cruz Biotechnology. Paraformaldehyde solution 4% PBS and UltraCruz™ Mounting Medium are from Santa Cruz Biotechnology.

### Generation of NCM460-NTR1 and NCM460-NTR1-AFTPH cells

NCM460 cells overexpressing NTR1 (NCM460-NTR1) were generated and maintained as described^8^. NCM460-NTR1-AFTPH cells were generated by transduction of lentivirus carrying full-length human AFTPH gene. The full-length human AFTPH gene was isolated from the original plasmid backbone pCMV6-Entry (Origene) by PCR. The purified AFTPH fragment was isolated from an agarose gel, digested with SpeI and Xho I and in inserted into the multiple cloning site (MCS) of pShuttle CMV-MCS-hrGFP-2 (Clontech) creating a 3× HA tag at the C-terminus. A PCR fragment of the full length AFTPH-HA was isolated from an agarose gel, digested with SpeI and ligated into the MCS of lentiviral backbone CMV-MCS-IRES-Puro to create the lentiviral vector CMV-AFTPH-HA-IRES Puro. Generation of lenti virus particles using the third generation packaging plasmids was as previously described^61^. Briefly, nonconfluent 293T cells were co-transfected with pMDLg/p RRE (gag/pol), pMD.G (encoding the VSV-G envelope), pRSV–REV and CMV-AFTPH-HA-IRES-Puro by the CaPO_4_-DNA coprecipitation method. Viral titer was determined by assessing viral p24 antigen concentration by ELISA (Coulter Immunetech, Miami, FL) and hereafter expressed as μg of p24 equivalent units per milliliter. After transduction, NCM460-NTR1-AFTPH cells were cultured in M3:D complete media supplemented with 10 μg/mL puromycin for selection. Expression of AFTPH in NCM460-NTR1-AFTPH cells was verified by RT-PCR.

### Biotinylation assay

Biotinylation of surface NTR1 was achieved by a modification of a previously described method^62^. NCM460-NTR1 cells were washed with PBS and labeled with 1 mg/mL EZ-link^®^ Sulfo-NHS-LC Biotin for 15 min at 4 °C. Unbound biotin was quenched with Tris-buffered saline (TBS) at 4 °C and cells were washed with HBSS, recovered in medium (37 °C for 30 min) and exposed to 100 nM NT for 1 hr at 37 °C. After washing with HBSS, 50 mM glutathione in HBSS was used to strip biotin from proteins remaining at the cell surface. Endocytosed NTR1 was allowed to recycle to plasma membrane in culture medium for 3 hr at 37 °C, biotin bound to recycled NTR1 was stripped, and unbound biotin was quenched by TBS. Cells were then lysed with 1% Triton X-100 in HBSS supplemented with protease inhibitor cocktail. NTR1 recycling was quantified using NTR1-specific ELISA. Antibodies against NTR1^14^ (2 μg/mL) were used to coat 96-well plate. The plate was blocked in PBS containing 3% bovine serum and 0.1% Triton X and equal amount of lysates were loaded to each well in triplicates and incubated for 16 hr at 4 °C. This was followed by washing with PBS containing 0.05% Tween-20 and incubation with IRDye® 800CW Streptavidin (1 hr, room temperature). After washing, the fluorescent intensity was read by Odyssey® CLx Infrared Imager (LI-COR Biosciences) and quantified by LI-COR® Image Studio (LI-COR Biosciences). Membrane-associated NTR1 was calculated by the equation 1/(biotinylated NTR1 after treatment/Total biotinylated NTR1).

### Messenger RNA and microRNA expression analysis

NCM460-NTR1 cells were washed once with ice-cold PBS after various treatments. Total RNA were extracted by TRIzol and reverse-transcribed into first strand cDNAs using random decamers and reverse transcriptase for mRNA expression analysis using TaqMan^®^ Universal PCR Master Mix (2×). Complementary DNAs for microRNA expression analysis were prepared with Universal cDNA Synthesis Kit II and ExiLENT SYBR^®^ Green master mix according to the manufacturer’s instructions.

### Cloning and site-directed mutagenesis

The miR-133α promoter-driven luciferase reporter construct (pGL3-miR-133α) was generated by ligating PCR products encoding the genomic region of 2000 bp upstream to miR-133α was Xho1/HindIII digest and pGL3-Basic. The primers used are i) miR-133α Xho1 F: ccgctcgagtttcaaagaaattagttcaaagcttaa; ii) miR-133α HindIII R: cccaagcttagtgctgctagtttggaatcc. The following site-directed mutagenesis was done using QuikChange II XL site-directed mutagenesis Kit according to the manufacturer’s instructions. ZEB1 binding site on the miR-133α promoter region (pGL3-miR-133α-ΔZEB1) was deleted using with primers: iii) miR133α-del216: gcacttaagtttaggcagtttaacacttctactagaaaaaa tgatgaaaaag; iv) miR133α-del216antisense: ctttttcatcattttttctagtagaagtgttaaactgcctaaacttaagtgc.

### Luciferase assays

Plasmids expressing AFTPH 3′UTR-driven luciferase, pGL3-miR-133α or pGL3-miR-133α-ΔZEB1 and pRL-TK (control) were transfected to NCM460-NTR1 cells using lipofectamine 2000. Two days after transfection NCM460-NTR1 cells were exposed to NT (1 hr), firefly and Renilla luciferase cell activities were detected using Dual-luciferase reporter assay system. The relative miR-133α promoter-driven luciferase activities were calculated by normalizing Firefly luciferase activity with that from Renilla luciferase.

### ChIP assay

NCM460-NTR1 cells were cross-linked and fixed after NT exposure for 1 hr using Pierce Agarose ChIP kit according to the manufacturer’s instructions. The ZEB1 binding region was immunoprecipitated by rabbit anti-ZEB1 antibody (Bethyl Laboratories). ZEB1 binding was quantified by real time PCR using a primer complementary to ZEB1 binding site in miR-133α promoter region (Applied Biosystems, assay ID: AJPACV3, Part no. 4441114).

### Localization of NTR1, EEA1, golgin 97 and TGN38

NCM460 cells overexpressing NTR1 (NCM460-NTR1)^8^ were transfected with as-miR-133α or si-AFTPH and their corresponding controls. NTR1 recycling studies were performed as previously described^14^. In brief, two days after transfection, NCM460-NTR1 cells were serum-fasted overnight and then exposed to 100 nM NT for 1 hr at 37 °C. NCM460-NTR1 cells were then washed with PBS twice and replenish with NT-free medium. Cells were fixed in PBS containing 4% (w/v) paraformeldehyde (pH 7.4, 20 min, 4 °C) and blocked in PBS containing 3% bovine serum and 0.1% Triton X. Cells were incubated (16 hr, 4 °C) with anti-NTR1 antibodies^14^ (2 μg/mL), washed and incubated (2 hr, room temperature) with bovine anti-goat-FITC (2 μg/mL). After washing with PBS, cells were mounted with UltraCruz™ Mounting medium.

Co-localization studies were performed as mentioned except that the fixed cells were incubated with two primary antibodies: anti-NTR1/anti-EEA1^63^, anti-AFTPH^19^/anti-EEA1, anti-AFTPH/anti-golgin97^63^; anti-AFTPH/anti-TGN38^64^ where appropriate. Bovine anti-rabbit- and bovine anti-mouse- IgG-R were used as secondary antibodies where appropriate.

The stained cells were imaged with a Zeiss LSM 510 Meta laser scanning confocal microscope using a Zeiss 63X Plan-Apo/1.4 oil immersion objective (numerical aperture 1.4). Five Z-stack images were captured (1024 × 1024 pixel resolution) per treatment. Co-localization of NTR1 and EEA1 was quantitatively analyzed using Zen Software (Zeiss). Average NTR1 intensity in cells under different treatments was quantified by the following equation: [NTR1 signal _(total − nucleus)_/Area _(total − nucleus)_].

### In-cell ELISA

In assays studying the expression levels of endosomal proteins, proteins related to TGN and NTR1, NCM460-NTR1 and NCM460-NTR1-AFTPH cells were seeded and fixed in 96-well culture plates. In assays studying NTR1 expression after recovery, NCM460-NTR1 and NCM460-NTR1-AFTPH cells were stimulated with NT (100 nM, 1 hr) and internalized NTR1 were allowed to recover in NT-free media in the presence of Bafilomycin A1 (100 nM), Brefeldin A (5 μg/mL) and DMSO (solvent control) for 3 hr before fixing. In-cell Western Blot was performed as the manufacturer’s instructions with modifications. Briefly, the cells were permeabilized with 0.1% Triton X-100 in 1 × PBS and blocked with Odyssey® Blocking Buffer (LI-COR) for 1 hr at room temperature. Antibodies against NTR1, AFTPH, Rab5a, Rab7 and Rab11 were diluted in the same blocking buffer (2 μg/mL) and incubated with the cells at 4 °C overnight. After washing with 0.1% Tween 20 in 1 × PBS, secondary antibodies against goat, rabbit and mouse were used as appropriate (2 μg/mL) and CellTag™ 700 Stain (LI-COR) were incubated with the cells at room temperature for 1 hr. The wells were then washed and blot dry and protein expression was detected with Odyssey® Classic Imager using Image Studio™ software.

### Statistical analysis

All results were derived from at least three sets of experiments, expressed as means ± SD and analyzed with Student’s t-Tests. In all statistical comparisons, *p* <  0.05 was used to indicate significant differences.

## Additional Information

**How to cite this article**: Law, I. K. M. *et al.* Neurotensin-induced miR-133α expression regulates neurotensin receptor 1 recycling through its downstream target aftiphilin. *Sci. Rep.* **6**, 22195; doi: 10.1038/srep22195 (2016).

## Supplementary Material

Supplementary Figure 1 2
