# Supplementary material for: Neurotensin-induced miR-133α expression regulates neurotensin receptor 1 recycling through its downstream target aftiphilin
Source: Sci Rep. 2016 Feb 23;6:22195. doi: 10.1038/srep22195 (PMC4763298; doi:10.1038/srep22195)
Supplement: Supplementary Figure 1 2 [file srep22195-s1.pdf]

Manuscript Title: Neurotensin-induced miR-133α expression regulates neurotensin receptor 1 recycling through its downstream target aftiphilin

Author List: Ivy Ka Man Law, Dane Jensen, Nigel W Bunnett, Charalabos Pothoulakis

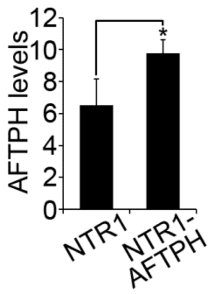

Supplementary Fig. 1. AFTPH was overexpressed in NCM460-NTR1-AFTPH cells. RT-PCR analysis showed AFTPH mRNA levels were increased in cells transduced with recombinant lentivirus expressing AFTPH. *p<0.05 when compared with NCM460-NTR1 cells.

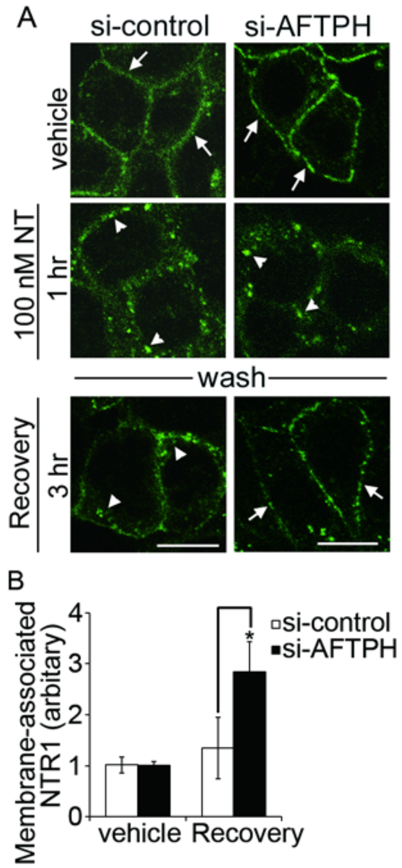

Supplementary Fig. 2. AFTPH gene silencing promoted NTR1 trafficking to plasma membrane during recovery in human colonic epithelial cells. (A) NCM460-NTR1 cells transfected with si-AFTPH and si-control were exposed to NT (100 nM, 1 hr), washed and replenished with NT-free media (recovery). Trafficking of NTR1 to plasma membrane was promoted in AFTPH knocked-down NCM460-NTR1 cells, as visualized using immunocytochemistry. (arrow: membrane-bound; arrowhead: vesicle-bound) Scale bars, 10 µM (B) AFTPH knock-down increased membrane-bound NTR1after recovery when compared with control cells, as measured in biotinylation assay. *$p<0.05$ when compared to si-control-transfected cells.
